# Effects of gut microbiota and probiotics on Alzheimer’s disease

**DOI:** 10.1515/tnsci-2020-0203

**Published:** 2021-12-27

**Authors:** Libing Guo, Jiaxin Xu, Yunhua Du, Weibo Wu, Wenjing Nie, Dongliang Zhang, Yuling Luo, Huixian Lu, Ming Lei, Songhua Xiao, Jun Liu

**Affiliations:** Department of Neurology, Foshan Third People’s Hospital, No. 102 Jinlan South Road, Foshan, Guangdong, China; Department of Neurology, Sun Yat-Sen Memorial Hospital, Sun Yat-Sen University, No. 107 Yanjiang West Road, Guangzhou, Guangdong, China; Guangdong Province Key Laboratory of Brain Function and Disease, Zhongshan School of Medicine, Sun Yat-Sen University, Guangzhou, China

**Keywords:** Alzheimer’s disease, gut microbiota, probiotics

## Abstract

Alzheimer’s disease (AD) is a progressive neurodegenerative disease with high morbidity, disability, and fatality rate, significantly increasing the global burden of public health. The failure in drug discovery over the past decades has stressed the urgency and importance of seeking new perspectives. Recently, gut microbiome (GM), with the ability to communicate with the brain bidirectionally through the microbiome–gut–brain axis, has attracted much attention in AD-related studies, owing to their strong associations with amyloids, systematic and focal inflammation, impairment of vascular homeostasis and gut barrier, mitochondrial dysfunction, etc., making the regulation of GM, specifically supplementation of probiotics a promising candidate for AD treatment. This article aims to review the leading-edge knowledge concerning potential roles of GM in AD pathogenesis and of probiotics in its treatment and prevention.

## 1 Introduction

The characteristic symptom of Alzheimer’s disease (AD) is cognitive deficiency in at least one cognitive areas, including memory recall, learning, concentration, etc., often accompanied by behavioral and psychological disorders [1]. It can be classified into different levels according to the severity, ranging from mild cognitive impairment (MCI) to dementia [2]. With high morbidity, disability, and fatality rate, AD is now a critical health problem that significantly increases global financial burden [3]. The prevalence of AD increases with age, reaching 23% in people over 86 years old, affecting millions of elderly people [4]. The fact that there is still no treatment that can effectively improve the crucial clinical outcomes of AD reveals that there is still a considerable gap in our knowledge of its complex pathogenesis, making a broader perspective urgent.

Gut microbiome (GM) is composed of the totality of microorganisms and their collective genetic materials in the gastrointestinal tract (GIT) [5]. GM has been found to play crucial roles in keeping homoeostasis and modulating functions of almost all major body systems, including the central nervous system (CNS) [6]. Research into the impacts of GM on the pathogenesis of AD has rapidly increased over the last decades. The discovery of the microbiota–gut–brain axis, a communication pathway between GM and the brain, has enhanced researchers’ confidence to regard the modification of GM as a potential approach to prevent or treat AD [7]. Thus, probiotics, live microbes which can positively alter the GM when taken in suitable amounts [8], are now regarded as potential candidates in the treatment of AD. In this article, we make a summary of the present knowledge on possible influences of gut microbiota on AD onset and progression and possible protective roles of probiotics against AD.

## 2 Gut microbiota and microbiota–gut–brain axis

There are over 1,000 species of microorganisms, including bacteria, archaea, yeasts, single-celled eukaryotes, helminth parasites, and viruses, with millions of genes totally in the human GIT [9]. There are various factors that can influence the GM population, such as modes of birth, age, diet, antibiotic exposure, and stress [10], some of which can also influence the pathogenesis of AD. A growing body of research has investigated the role of GM in human growth and health maintenance. In addition to interacting with human immune system via toll-like receptors (TLRs), GM can also act as a biological barrier to prevent abnormal microbiota from invading [11] and even participate in the maturation process of our immune system [12]. GM have been found to affect maturation and function of microglia [13,14]. As for metabolism, GM could play different roles, ranging from regulating the glucose and lipid homeostasis [15] to immunomodulatory effects [16]. Notably, there are some important metabolites produced by the microbiota, which include neurotransmitters such as γ-aminobutyric acid (GABA), serotonin (5-HT) and dopamine, and short-chain fatty acids (SCFAs), which have been found to be associated with changes of host brain functions [17,18].

GM communicates with brain bidirectionally via the microbiome–gut–brain axis involving various routes, including the immune system, vascular system, enteric nervous system, and the vagus nerve [19]. The lack of GM in germ-free (GF) mice could lead to multiple alterations in the nervous system, such as altered concentration of various neurotransmitters (5-HT, dopamine, GABA, etc.) and modulation of synaptic plasticity and transmission, leading to behavioral and emotional abnormalities [20]. Thus, the axis has received considerable attention in studies on neurological disorders, including AD.

## 3 Gut microbiota and AD

AD is a progressive neurodegenerative disorder, with extracellular amyloid-β (Aβ) plaques and intracellular neurofibrillary tangles (NFTs) made of hyperphosphorylated tau as its pathological hallmarks [21]. In addition, chronic neuroinflammation led by excessive microglial activation, astrocyte reactivity, and increased load of proinflammatory cytokines and chemokines has also been found to play a vital role in AD pathology [22,23,24]. Neuroinflammation can not only precede Aβ and tau aggregation but also influence Aβ production, aggregation, and clearance [13], whereas Aβ complexes can bind to pattern recognition receptors expressed in microglia and astrocytes and contribute to neuroinflammation dysregulation and generation of reactive oxygen species (ROS), leading to death of neurons and glial cells [22]. Vascular risk factors such as hypertension, atherosclerosis, and diabetes can also increase the risk of AD. Vascular lesions and dysfunction are also now regarded as important early events in AD pathophysiology [25,26]. The etiology and pathogenesis of AD are still not fully understood, leading to the failure in clinical trials of drugs targeting toward generally acknowledged molecules, especially Aβ. AD is now regarded as a multifaceted disorder influenced by various risk factors such as age, cerebrovascular risk factors, psychogenic diseases [27], and GM.

Increasing evidence indicates a strong association between GM and AD. GM changes have been noticed in fecal samples from patients with AD [6] and transgenic AD mice [28,29]. Studies on the mechanisms behind this illuminating association may provide new viewpoints on the pathogenesis and intervention of AD.

### 3.1 Gut microbiota and amyloid-related pathogenesis

Amyloids can be secreted by various members of human GM, such as *E. coli*, *S. typhimurium*, *Bacillus subtilis*, *Staphylococcus aureus*, *Pseudomonas fluorescens*, etc. [30]. GM-derived and human amyloids are involved in complex interactions with the immune system. Although microbial amyloids like CsgA do not have the same amino acid sequences as human Aβ_1-42_, they still contain similar pathogen-associated molecular patterns (PAMPs) and thus could interact with the same TLR2, inducing proinflammatory interleukin IL-17A and potent inflammatory mediators such as IL-22, followed by the activation of NF-κB signaling pathway and cyclooxygenase-2 (COX-2) [31,32,33]. Bacterial amyloid proteins are also found to promote misfolding and aggregation of neuronal Aβ peptides through cross-seeding [30,34,35]. Recent evidence indicates that Aβ can also act as an antimicrobial peptide that participates in host immune response to microbes through fibrillation, entrapping pathogens, and disrupting cell membranes [36].

### 3.2 Gut microbiota dysbiosis and inflammation-driven pathogenesis

Increasing evidence suggests that the association between dysregulation of GM and altered inflammatory states might explain the influences of GM in the initiation or exacerbation of AD. Cattaneo et al. investigated the GM taxa changes in patients with CI and brain amyloidosis, showing an increased abundance of *Escherichia*/*Shigella*, a proinflammatory taxon and a decreased abundance of *E. rectale*, an anti-inflammatory taxon, which was found associated with changes of peripheral inflammation [37]. The GF condition or chronic antibiotic treatment in AD mice could result in reduced insoluble amyloid plaques and suppressed neuroinflammation, attenuated microglia, and astrocyte aggregation around the plaques in the hippocampus [28,38,39].

**Figure 1:**
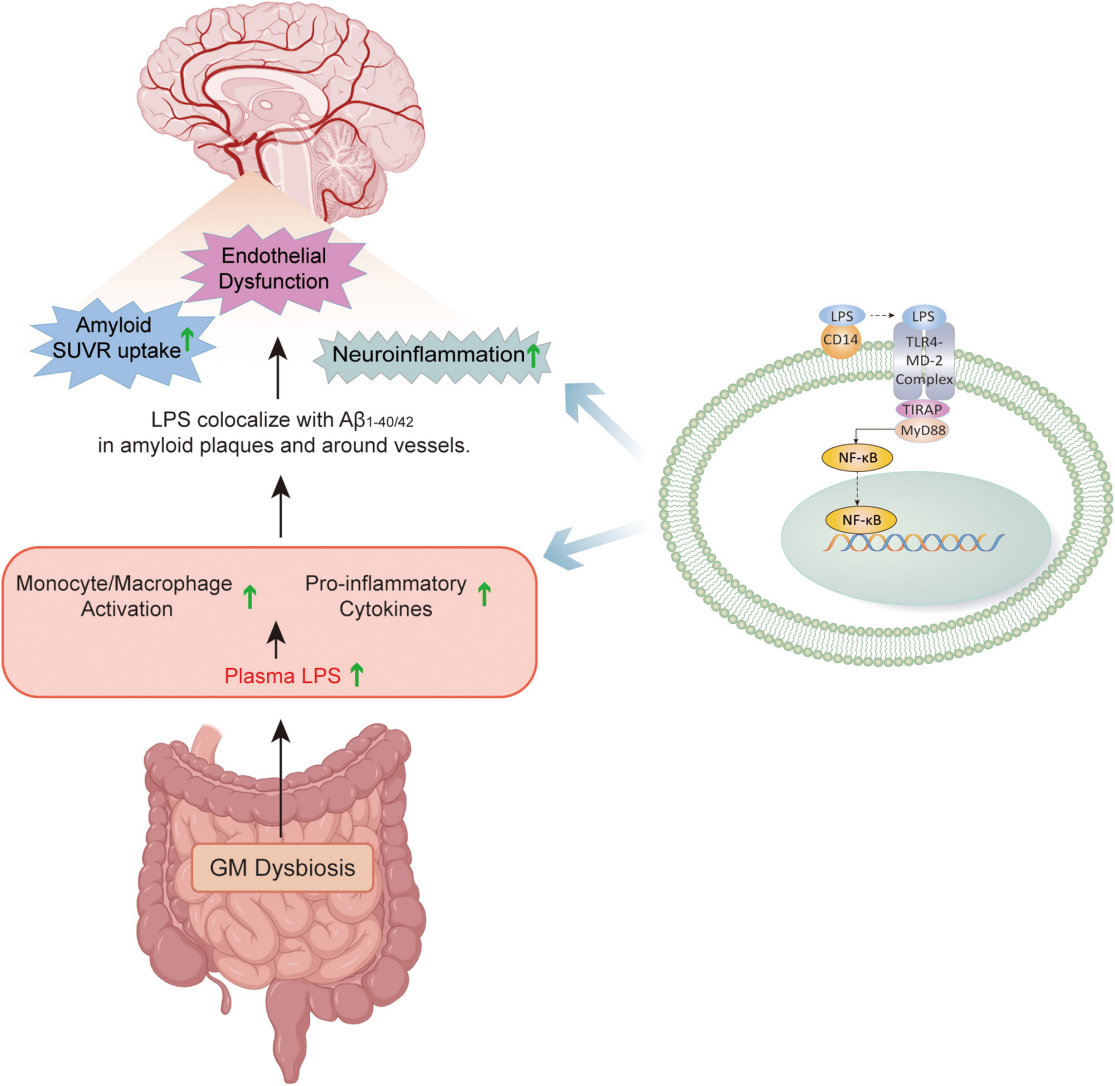
Proposed mechanism of LPS affecting the pathogenensis of AD. AD-related GM dysbiosis contributes to an increased level of plasma LPS, which promotes blood monocyte/macrophage activation and the secretion of pro-inflammatory cytokines mainly through NF-κB pathway. LPS can also cross the BBB, promote neuroinflammation and colocalize with Aβ1-40/42 in amyloid plaques and around vessels in the brain, possibly affecting Aβ pathology and endothelial function.

Lipopolysaccharide (LPS), which is located in the outer membrane of Gram-negative bacteria, is considered as an important mediator between GM dysbiosis and AD pathology (Figure 1). It can initiate potent immune responses by interacting with CD14 and the TLR4-MD-2 complex of immune cells. TLR4 can interact with TIRAP and MyD88 and then induce the activation of NF-κB, a pro-inflammatory transcription factor which is known for triggering pathogenic pathways in AD by promoting the secretion of proinflammatory cytokines [40–43]. Zhang et al. reported that the level of plasma LPS was elevated in AD patients, which was correlated positively with the level of blood monocyte/macrophage activation [44]. LPS was also detected in the hippocampus and superior temporal lobe neocortex in patients with AD [45]. LPS was found to colocalize with Aβ_1-40/42_ in amyloid plaques and around vessels [46]. Interestingly, Marizzoni et al. reported that amyloid standardized uptake value ratio uptake was positively associated with the levels of blood LPS, proinflammatory cytokines, and endothelial dysfunction [47].

### 3.3 Gut microbiota dysbiosis impairs vascular homeostasis and gut barrier

It is well established that the impairment of vascular homeostasis plays an essential role in the development of AD [25]. Furthermore, the invasion of GM and their metabolites into the brain depends heavily on the permeability of the blood–brain barrier (BBB) and the gut epithelial barrier, which can also be affected by GM dysbiosis [48]. Transplant of the fecal microbiota from pathogen-free adult mice was found to upregulate the expression of tight junction proteins and decrease BBB permeability in GF adult mice [49]. Engen et al. reported a link between increased proinflammatory GM with impaired gut barrier function [50]. Bacterial products such as LPS and amyloids have been found to impair BBB through triggering chronic neuroinflammatory responses [51]. GM dysbiosis can also affect trimethylamine oxide levels that regulate vascular microRNA, leading to atherosclerosis, a common risk factor for AD [52].

### 3.4 Gut microbiota dysbiosis causes mitochondrial dysfunction

Mitochondrial dysfunction, existing as an early event of AD, can lead to decreased energy metabolism and oxidative phosphorylation of key enzymes. It is also found to contribute to neuronal apoptosis and calcium homeostasis disorders [53]. The imbalance of mitochondrial/cellular antioxidant system may lead to the decrease of PTEN-induced putative kinase 1 (PINK1) expression, resulting in reduced ATP production and abnormal brain metabolism and eventually leading to cognitive dysfunction [54,55]. Due to the symbiotic relationship between mitochondrial metabolic diversity and primitive aerobic and anaerobic bacteria, it can provide energy for the host under both aerobic and anaerobic conditions. The differences in bacterial composition and the changes of metabolite production caused by GM dysbiosis may lead to mitochondrial dysfunction, thus increasing oxidative stress and inflammatory response of the host [56]. Therefore, the prevention of mitochondrial dysfunction and reduction of oxidative stress may be promising methods for the prevention or alleviation of cognitive dysfunction.

### 3.5 Other pathways

Glutamate acts as a major excitatory neurotransmitter involved in the process of memory and learning [57]. Recent studies have shown that GM including *Bacteroides vulgatus* and *Campylobacter jejuni* could influence the metabolism of glutamate. Moreover, d-glutamate metabolized by GM may interact with the glutamate N-methyl-D-aspartate-receptor and influence cognitive function in AD patients [58]. Brain-derived neurotrophic factor (BDNF) is a major protective factor fighting neurodegeneration, particularly in AD [59]. It is proposed that GM are able to affect the level of brain BDNF as decreased BDNF level and abnormal behavior in GF mice could be normalized after colonized with probiotic administration [60]. SCFAs, including acetate, butyrate, and propionate, can act as modulators of both peripheral NS and CNS based on their ability to cross and even influence gut barrier and BBB. [61] SCFAs can also regulate microglia homeostasis as there was a microglia defect in the mice that lack the free fatty acid receptor 2 (FFAR2), one of the SCFA receptors. A similar change occurred under a GF condition [14]. Administration of sodium butyrate at an advanced stage of progression was reported to improve memory in AD mice, possibly through increasing expression of learning-associated genes and restoring histone acetylation [62]. Butyrate also acts as a major energy source of intestinal cells and is able to make mitochondrial respiration rate and ATP production higher [63,64].

## 4 Probiotics as potential therapeutics for AD

Accumulated clinical evidence has implied therapeutic potential for probiotics in AD through various mechanisms. A meta-analysis conducted in 2019 indicated that probiotics could improve cognitive performance in MCI and AD patients, possibly due to their anti-inflammatory and antioxidative effects [65]. In a randomized, double-blind and controlled trial, probiotic milk containing *Lactobacillus acidophilus*, *Lactobacillus casei*, *Bifidobacterium bifidum*, and *Lactobacillus fermentum* for 12 weeks was reported to significantly improve cognitive performance in AD patients. Further assessment showed positive influence of probiotics on markers of insulin resistance, plasma level of malondialdehyde, and serum levels of high-sensitivity C-reactive protein, triglyceride, and very low density lipoprotein, whereas it shows no improvement in fasting plasma glucose, other lipid profiles, and biomarkers of inflammation and oxidative stress [66] Large-scale, long-period, randomized controlled trials are needed for more reliable evidence.

Attempts have been made to unravel the effects of probiotics on AD pathology and pathophysiology. Previous studies have implied significant anti-inflammatory effects and cognitive benefits of various probiotics [67–70]. Bonfili et al. reported that the administration of SLAB51 mixture (a formulation including *Streptococcus thermophilus*, bifidobacteria, and lactobacilli) could positively influence levels of plasma inflammatory cytokines, restore the impaired ubiquitin proteasome system and autophagy, reduce Aβ load, and ameliorate cortical atrophy in AD mice [69]. Previous studies have also demonstrated respective anti-inflammatory effects of *Lactobacillus johnsonii* and *Bifidobacterium infantis* through modulating the kynurenine pathway of tryptophan degradation [71,72]. Kobayashi et al. found that *Bifidobacterium breve* A1 could restore the Aβ-induced changes in the expression of inflammation and immune-reactive genes in the hippocampus. They also noticed an increase in the plasma level of acetate, which could partially alleviate behavioral deficits in AD model mice [68]. *Lactobacillus plantarum C29* was reported to regulate microglia activation, suppress NF-κB activation, and reduce Aβ deposition in the brain of 5xFAD transgenic mice [70].

Despite current supportive evidence on therapeutic potential of probiotics, more studies are warranted to develop an effective and safe probiotic formulation for AD prevention or treatment. In fact, no probiotic formulation has been approved as a therapeutic modality by major medical regulatory authorities due to the lack of better evidence-based proof of their health-promoting ability and adverse effects [73]. It is important to note that probiotic intake may cause serious adverse events such as sepsis, especially among vulnerable population including the elderly, critically ill, and immunocompromised patients [61,73]. Interestingly, several studies implied that probiotics use after antibiotic treatment could increase the risk of communicable diseases by inducing a persistent long-term dysbiosis [73–77]. Another noteworthy adverse event of probiotics is serotonin syndrome, which is often caused by selective serotonin reuptake inhibitor (SSRI) use in patients with depression. Tryptophan-metabolizing probiotics alone can rarely trigger the syndrome while its combination with potent SSRI can significantly increase the risk [61,78]. As people with or at risk of AD are always elderly and sometimes with depression, probiotics should be administered much more carefully among these people.

## 5 Conclusion

The bottleneck in current AD therapy is the poor understanding of AD pathogenesis, for which the studies on the association between GM and AD may open new horizons. GM dysbiosis has been found correlated with various AD biomarkers, thus correcting it through supplementation of probiotics may be a potential method to treat AD. Several well-designed clinical and mechanistic studies are warranted to further elucidate the underlying mechanisms and develop an effective and safe probiotic formulation for AD prevention and treatment. Emerging novel probiotic drugs are being studied and designed to reduce related risks and enhance their therapeutic ability. While there are many challenges that remain, researchers around the world should still be optimistic about this flourishing field linking AD to gut microbiota.
